# Pharmacological and Genetic Inhibition of HDAC4 Alleviates Renal Injury and Fibrosis in Mice

**DOI:** 10.3389/fphar.2022.929334

**Published:** 2022-06-28

**Authors:** Fengchen Shen, Xiying Hou, Tingting Li, Jianjun Yu, Huizhen Chen, Na Liu, Andong Qiu, Shougang Zhuang

**Affiliations:** ^1^ Department of Nephrology, Shanghai East Hospital, Tongji University School of Medicine, Shanghai, China; ^2^ School of Life Science and Technology, Advanced Institute of Translational Medicine, Tongji University, Shanghai, China; ^3^ Department of Medicine, Rhode Island Hospital and Alpert Medical School, Brown University, Providence, RI, United States

**Keywords:** histone deacetylase 4, renal fibrosis, tasquinimod, knockout mice, unilateral ureteral obstruction, epithelial–mesenchymal transformation

## Abstract

Histone deacetylase 4 (HDAC4) has been shown to be involved in cell proliferation, differentiation, and migration and is associated with a variety of cancers. However, the role of HDAC4 in renal fibrogenesis and its mechanisms are unclear. We assessed the role of HDAC4 and possible mechanisms of fibrosis in a murine model of kidney injury induced by unilateral ureteral obstruction (UUO) using tasquinimod, a highly selective HDAC4 inhibitor, and knockout mice with depletion of HDAC4 in renal tubular cells. UUO injury resulted in increased expression of HDAC4 and fibrotic proteins fibronectin and α-smooth muscle actin, while treatment with tasquinimod or knockout of HDAC4 significantly reduced their expression. Pharmacological and genetic inhibition of HDAC4 also decreased tubular epithelial cell arrest in the G2/M phase of the cell cycle, expression of transforming growth factor-β_1_ and phosphorylation of Smad3, signal transducer and activator of transcription 3, and extracellular signal-regulated kinase 1/2 in the injured kidney. Moreover, tasquinimod treatment or HDAC4 deletion inhibited UUO-induced renal tubular cell injury and apoptosis as indicated by reduced expression of neutrophil gelatinase–associated lipocalin, Bax, and inhibition of caspase-3. Finally, administration of tasquinimod or knockdown of HDAC4 prevented injury-related repression of Klotho, a renoprotective protein. Our results indicate that HDAC4 is critically involved in renal tubular injury and fibrosis and suggest that HDAC4 is a potential therapeutic target for treatment of chronic fibrotic kidney disease.

## Introduction

Chronic kidney disease (CKD) is a common health concern worldwide, manifesting as chronic kidney structural and functional disorders. The global prevalence of CKD is 13.4%, of which 10.6% of CKD patients are in stages 3–5 (Hill et al., 2016). Renal fibrosis is the major pathological process for CKD to progress to end-stage renal disease (ESRD). Although the application of renal replacement therapies such as dialysis and kidney transplantation can improve the outcome of patients with ESRD, there is still a lack of effective approaches to halt the progression of renal fibrosis. Therefore, elucidating the mechanism of renal fibrogenesis will aid in identifying novel therapeutic targets for chronic renal fibrotic diseases.

Emerging evidence indicates that renal fibrosis is a process involved in multiple pathophysiological changes, in particular, renal tubular injury. When renal tubular epithelial cells are severely injured, some die by apoptosis and necrosis, while surviving tubular cells undergo maladaptive repair, leading to a partial epithelial–mesenchymal transition (pEMT) and arrest at the G_2_/M phase of the cell cycle (Macedo et al., 2008). This phenotype of cells acquires the ability to produce pro-inflammatory/pro-fibrotic factors. These factors are then released into the renal interstitium to stimulate activation/proliferation of renal interstitial fibroblasts and subsequent deposition of extracellular matrix (ECM) proteins such as fibronectin and collagen I and III (Y. Liu, 2011; Iwano et al., 2002). Among the pro-inflammatory/pro-fibrotic factors released, transforming growth factor-β1 (TGF-β_1_) is recognized as the most potent profibrotic factor, which not only activates renal interstitial fibroblasts but also induces the EMT of renal epithelial cells. Many studies have documented that TGF-β_1_ stimulates renal fibrosis primarily through activation of the Smad3 pathway (Masszi et al., 2004; Masszi et al., 2010; Charbonney et al., 2011). Chronic injury and TGF-β_1_ can also initiate the fibrotic response through some non-Smad signaling pathways, including activation of signal transducer and activator of transcription 3 (STAT3) (Pang et al., 2010; Matsui and Meldrum, 2012; Bienaime et al., 2016) and extracellular signal–regulated kinase ½ (ERK1/2) (Andrikopoulos et al., 2019; J.; Chen et al., 2012; Rhyu et al., 2005).

Histone acetylation, one of the protein epigenetic modifications, plays a crucial role in renal fibrosis by regulating gene expression and catalyzing the activation of profibrotic signaling pathways (Pang et al., 2009; Tang et al., 2013; Zhang et al., 2020). It usually occurs on the specific lysine residue at the amino-terminal of histone and non-histone proteins and is mutually regulated by histone acetyltransferase and histone deacetylase (HDAC). HDAC is a family with 18 members divided into four categories. Class I HDACs include HDAC1, 2, 3, and 8; Class II HDACs are divided into two subclasses: Class IIa (HDAC4, 5, 7, and 9) and class IIb (HDAC6, 10). Class III HDAC contains SIRT1-7, while class IV HDAC only has HDAC11. Among them, HDAC4 has attracted attention because of its functional involvement in many physiological and pathological conditions. For example, HDAC4 activation contributes to retinal development and neuronal activation (B. Chen and Cepko, 2009) and is associated with the development of some chronic diseases such as hypertrophic cardiomyopathy (Backs et al., 2006), ataxia telangiectasia (Li et al., 2012), and hepatic fibrosis (Han et al., 2017). HDAC4 is also involved in tumorigeneses of multiple cancers, including nasopharyngeal carcinoma, colon cancer, and multiple myeloma (Wilson et al., 2008; Amodio et al., 2016; Cheng et al., 2021). Interestingly, the administration of tasquinimod, a specific inhibitor of HDAC4, can effectively inhibit the growth and metastasis of tumor cells *in vitro* and *in vivo* (Williamson et al., 2013; Raymond et al., 2014). This inhibitor has entered phase III clinical trials for the treatment of prostate cancer (Gupta et al., 2014; Brower, 2016).

To date, the role and mechanisms of HDAC4 action in renal fibrosis remain undefined. In this study, we utilized tasquinimod and knockout mice of HDAC4 to investigate the role of HDAC4 in the development of renal fibrosis and the mechanisms involved in a murine model of renal fibrosis induced by unilateral ureteral obstruction (UUO). We demonstrated that pharmacological and genetic inhibition of HDAC4 largely attenuates renal fibrosis by suppressing several profibrotic signaling pathways, reducing renal injury, and retaining the Klotho expression.

## Materials and Methods

### Chemical and Antibodies

Tasquinimod was purchased from Selleckchem (Houston, TX, United States). Antibodies to Smad3, p-Smad3, acetyl-H3, p-H3, H3, GAPDH, p-STAT3, STAT3, p-ERK, ERK, anti–cleaved caspase-3, HDAC4, and E-cadherin were purchased from Cell signaling Technologies (Danvers, MA, United States). Antibodies to Klotho and Bax were purchased from Santa Cruz Biotechnology (Dallas, Texas, United States). Antibody to TGF-β_1_ was purchased from Abcam (Cambridge, United Kingdom). Antibody to NGAL was purchased from R&D Systems (Minnesota, United States). Antibodies to α-SMA and fibronectin were purchased from Absin Bioscience Inc (Shanghai, China).

### Animals Models of Renal Fibrosis and Treatment

C57BL/6 male mice were purchased from Shanghai JISJIE Experimental Animal Co., LTD. (Shanghai, China). HDAC4^fl/fl^ and Cdh16-Cre mice (Cdh16-CreER+/-) were purchased from Shanghai Nanfang Model Biotechnology Co., LTD. (Shanghai, China). The HDAC4 knockout mice were generated by breeding HDAC4^fl/fl^ with Cdh16-Cre ± mice to obtain Cdh16-Cre^+^: HDAC4^fl/fl^ mice (HDAC4-KO) and Cdh16-Cre^-^: HDAC4^fl/fl^ mice (HDAC4-WT). All mice were fed with water and food in a 12-h light–dark cycle at the Animal Experimental Center of Tongji University until they were 8 weeks old and weighed 20–25 g. Laboratory animals and animal experiments are reviewed and approved by the Animal Care and Use Committee of Tongji University.

A murine unilateral ureteral obstruction model was created according to established methods (Pang et al., 2009). Briefly, the mice underwent abdominal surgery with a 1-cm dorsal incision, and the left ureter was separated and ligated near the renal pelvis. The contralateral kidney was used as a control. To examine its effect on renal fibrosis, tasquinimod was dissolved in 5% CMC-Na and injected intraperitoneally 24 h after UUO at a dose of 25 mg/kg/d for 6 consecutive days. C57BL/6 male mice were used in this study. They were randomly divided into four groups with six mice in each group: (ⅰ) sham group; (ⅱ) tasquinimod-treated sham group; (ⅲ) UUO group; (ⅳ) tasquinimod-treated UUO group. The HDAC4 knockout mice were also divided into four groups: (ⅰ) Cdh16-Cre^-^: HDAC4^fl/fl^-Sham group; (ⅱ) Cdh16-Cre^-^HDAC4^fl/fl^-Sham group; (ⅲ) Cdh16-Cre^-^: HDAC4^fl/fl^-UUO group; (ⅳ) Cdh16-Cre^+^HDAC4^fl/fl^-UUO group. All mice were euthanized 7 days after surgery, and kidneys were removed for analysis.

### Western Blot Analysis

Total proteins were extracted from the kidneys, and concentrations were measured using a BCA protein assay kit (Beyotime, Shanghai, China). The proteins were then separated by sodium dodecyl sulfate-polyacrylamide gel electrophoresis (SDS-PAGE). After SDS-PAGE, the proteins were electro-transferred and bound with PVDF membranes (Millipore, Bedford, MA, United States). The membranes were blocked with 5% skim milk and then incubated overnight with a primary antibody on a shaker in a 4°C refrigerator, followed by 1 h of incubation with a secondary antibody. Protein bands were visualized by an enhanced chemiluminescence reagent and exposed using BioImaging Systems (UVP, Upland, CA, United States). Relative protein levels were quantified using ImageJ software (National Institutes of Health, Montgomery, MD, United States).

### Pathological Assessment

The kidney tissue was fixed with formalin, embedded in paraffin, and sliced to about 3–5 µM thickness. Masson’s trichrome staining was carried out by the manufacturer’s instructions to assess fibrosis. The ECM area staining positive for fibrosis was observed under a light microscope, and the average value of 10 independent measurements was calculated by ImageJ.

### Immunofluorescence Staining

Immunofluorescence was performed on kidney tissues as described in our previous study (Pang et al., 2009). Kidney sections were incubated with vimentin, HDAC4, and Klotho primary antibodies, followed by labeling with different fluorescent-labeled secondary antibodies.

### Statistical Analysis

Data depicted in graphs represent the means ± SEM for each group. Intergroup comparison was made using one-way ANOVA. Multiple means were compared using Tukey’s test. The differences between the two groups were determined by Student’s t-test. Statistically significant differences between mean values were marked in each graph. *p* < 0.05 was considered significant.

## Results

### Pharmacological Inhibition of HDAC4 by Tasquinimod Alleviates Renal Fibrosis Following Ureteral Obstruction

**FIGURE 1:**
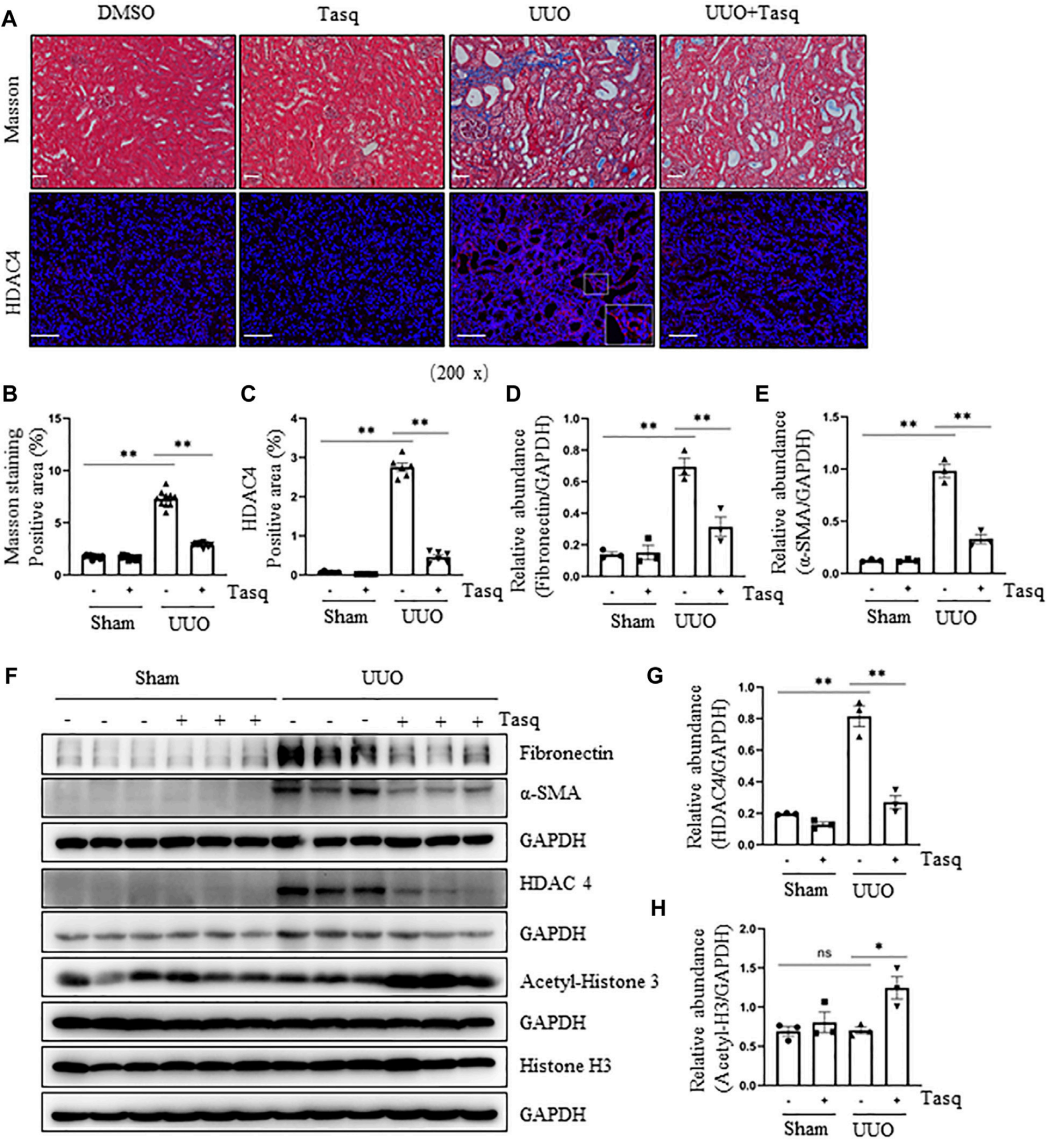
Inhibition of HDAC4 by tasquinimod (Tasq) alleviates renal fibrosis after ureteral obstruction. **(A)** Micrographs of Masson’s trichrome staining (Original magnification ×200, Scale bar = 50 μm). Micrographs of HDAC4 immunofluorescence staining; the lower right of HDAC4/UUO is a micrograph twice the size of the original partial position (original magnification ×200, Scale bar = 100 μm). **(B)** Graph shows the positive area of the Masson-positive tubulointerstitial fibrosis (blue) from 10 random fields of kidney samples from three mice. **(C)** HDAC4 staining shows quantitative data. Data are presented as average SE (*n* = 6). Scale bar = 100 μm. **(F)** The kidney tissue lysates were subjected to immunoblot analysis with antibodies against fibronectin, α-SMA, HDAC4, acetyl-histone 3, histone 3, or GAPDH. Expression levels of fibronectin **(D)**, α-SMA **(E)**, and HDAC4 **(G)** were quantified by densitometry and normalized using GAPDH. Acetyl-histone 3 **(H)** was normalized with histone 3. Values are the means ± SEM of three samples. NS means no statistical significance; *p < 0.05; **p < 0.01.

Our recent studies show that blocking Class IIa HDACs with MC1568 effectively inhibits the UUO-induced renal fibrosis (Xiong et al., 2019) and that HDAC4 is the most expressed class IIa isoform in the kidney. To specifically determine the role of HDAC4 in the development of renal fibrosis and assess the therapeutic effect of HDAC4 inhibition, we established a mouse model of the UUO-induced renal interstitial fibrosis and then injected tasquinimod 24 h *via* I.P after surgery, followed by daily injections for 6 constitutive days. Masson’s staining showed that ECM deposition in the renal interstitium in the UUO injured kidney was largely attenuated by tasquinimod compared to UUO alone (Figures 1A,B). Immunoblot analysis demonstrated reduced expression of fibronectin, an ECM protein, and α-smooth muscle actin (α-SMA), a hallmark of myofibroblasts, in UUO mice treated with tasquinimod compared to UUO alone (Figures 1D–F). As expected, the expression level of HDAC4 was increased in injured kidneys and decreased in kidneys treated with the inhibitor, as demonstrated by both immunofluorescence staining and immunoblot analysis (Figures 1A,C,F,G). In line with our previous studies, HDAC4 was primarily expressed in renal tubules but not in the interstitium of UUO injured kidneys (Xiong et al., 2019). Administration of tasquinimod increased the expression level of acetyl-histone H3 (Figures 1F,H) but did not alter the level of total histone H3. Furthermore, UUO affected the expression of neither acetylated histone H3 nor its total levels.

These results suggest that HDAC4 plays an essential role in mediating renal fibrosis, and pharmacological inhibition of HDAC4 with tasquinimod can effectively suppress HDAC4 expression and reduce renal fibrosis.

### Specific Deletion of HDAC4 in Renal Tubules Alleviates Renal Fibrosis Following Ureteral Obstruction

**FIGURE 2:**
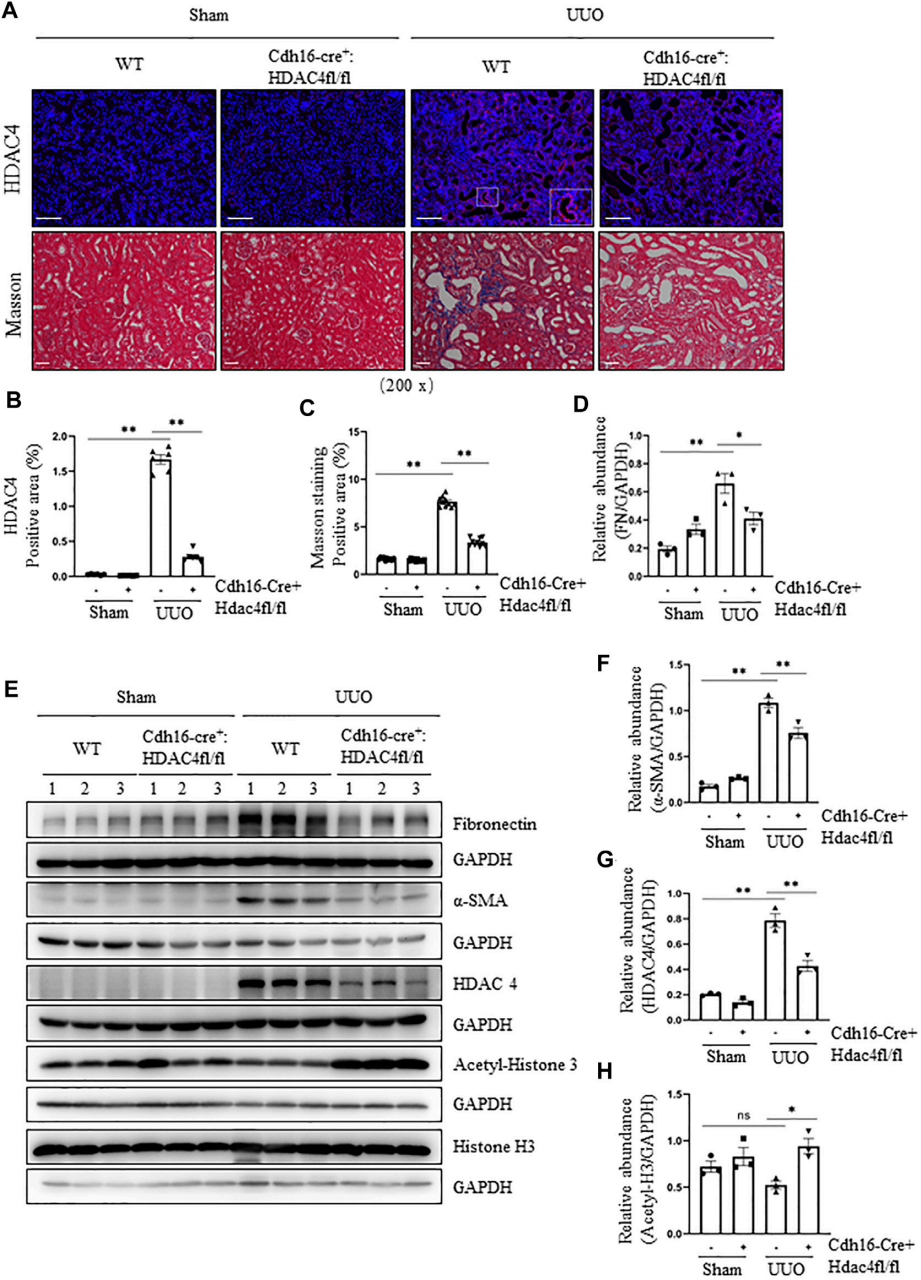
Specific deletion of HDAC4 in renal tubules alleviates renal fibrosis after ureteral obstruction. Cdh16-Cre^-^: HDAC4^fl/fl^ (HDAC4-WT) and Cdh16-Cre^+^: HDAC4^fl/fl^ (HDAC4-KO) mice were sacrificed 7 days after sham operation or UUO. **(A)** Micrographs of HDAC4 immunofluorescence staining; the box in lower right micrograph in HDAC4/WT/UUO is twice the size of the original partial position (original magnification ×200, Scale bar = 100 μm). Micrographs of Masson’s staining (original magnification ×200, Scale bar = 50 μm). **(B)** HDAC4 staining shows quantitative data. Data are presented as average SE (*n* = 6). Scale bar = 100 μm. **(C)** Graph shows the positive area of the Masson-positive tubulointerstitial fibrosis (blue) from 10 random fields of kidney samples from three mice **(C)** Graph shows the positive area of the Masson-positive tubulointerstitial fibrosis (blue) from 10 random fields of kidney samples from three mice. **(E)** Kidney tissue lysates were subjected to immunoblot analysis with antibodies against fibronectin, α-SMA, HDAC4, acetyl-histone 3, histone 3, or GAPDH. Expression levels of fibronectin **(D)**, α-SMA **(F)**, and HDAC4 **(G)** were quantified by densitometry and normalized using GAPDH. Acetyl-histone 3 **(H)** was normalized with histone 3. Values are the means ± SEM of three samples. NS means no statistical significance; *p < 0.05; **p < 0.01.

To confirm the role of HDAC4 in renal fibrosis, we further examined the genetic inhibition of HDAC4 on the expression of the ECM proteins and the activation of renal fibroblasts in mice following UUO injury. Cdh16-Cre^+^: HDAC4^fl/fl^ mice (HDAC4-KO)and Cdh16-Cre^-^: HDAC4^fl/fl^ mice (HDAC4-WT) mice were generated by crossing Cdh16-Cre^+/-^ and HDAC4^fl/fl^ mice. Previously, Cdh16 has been identified to be predominately expressed in collecting ducts and moderately expressed in distal convoluted tubules (Shao et al., 2002a; Shao et al., 2002b), implicating deletion of HDAC4 in these tubular segments of the kidney in Cdh16-Cre^-^: HDAC4^fl/fl^ mice. This was demonstrated by the absence of HDAC4 in tubules of the HDAC4-KO mice by immunofluorescence staining (Figures 2A,C). Immunoblot analysis confirmed a reduction of HDAC4 expression in the HDAC4-KO mice relative to that in the kidney of WT mice. Notably, a small amount of HDAC4 was still present in the kidney of the HDAC KO mice, indicative of a basal level of HDAC4 in other tubular segments and/or other cell types in the kidneys (Figures 2E,G). In contrast to the expression profile of HDAC4, renal acetyl-histone H3 was elevated in the Cdh16-Cre^-^: HDAC4^fl/fl^ mice (Figures 2E,H), suggesting that HDAC4 plays a negative role in the regulation of protein acetylation in the kidney after UUO injury.

We also examined the fibrotic changes in the HDAC4-WT and HDAC4-KO mice. Masson’s staining showed that there was no ECM deposition in the renal interstitium of normal HDAC4-WT and HDAC4-KO mice; the ECM was increased in WT mice subjected to UUO but decreased in the HDAC4-KO mice under the same pathological condition (Figures 2A,B). Moreover, the HDAC4-KO mouse kidneys had a lower expression level of fibronectin and α-SMA than the HDAC4-WT mouse kidneys after UUO injury (Figures 2D–F). These data are consistent with our findings using tasquinimod, confirming the importance of HDAC4 in regulating renal fibrogenesis.

### Pharmacological and Genetic Inhibition of HDAC4 Attenuates the pEMT and G_2_/M Cell Cycle Arrest After Ureteral Obstruction

**FIGURE 3:**
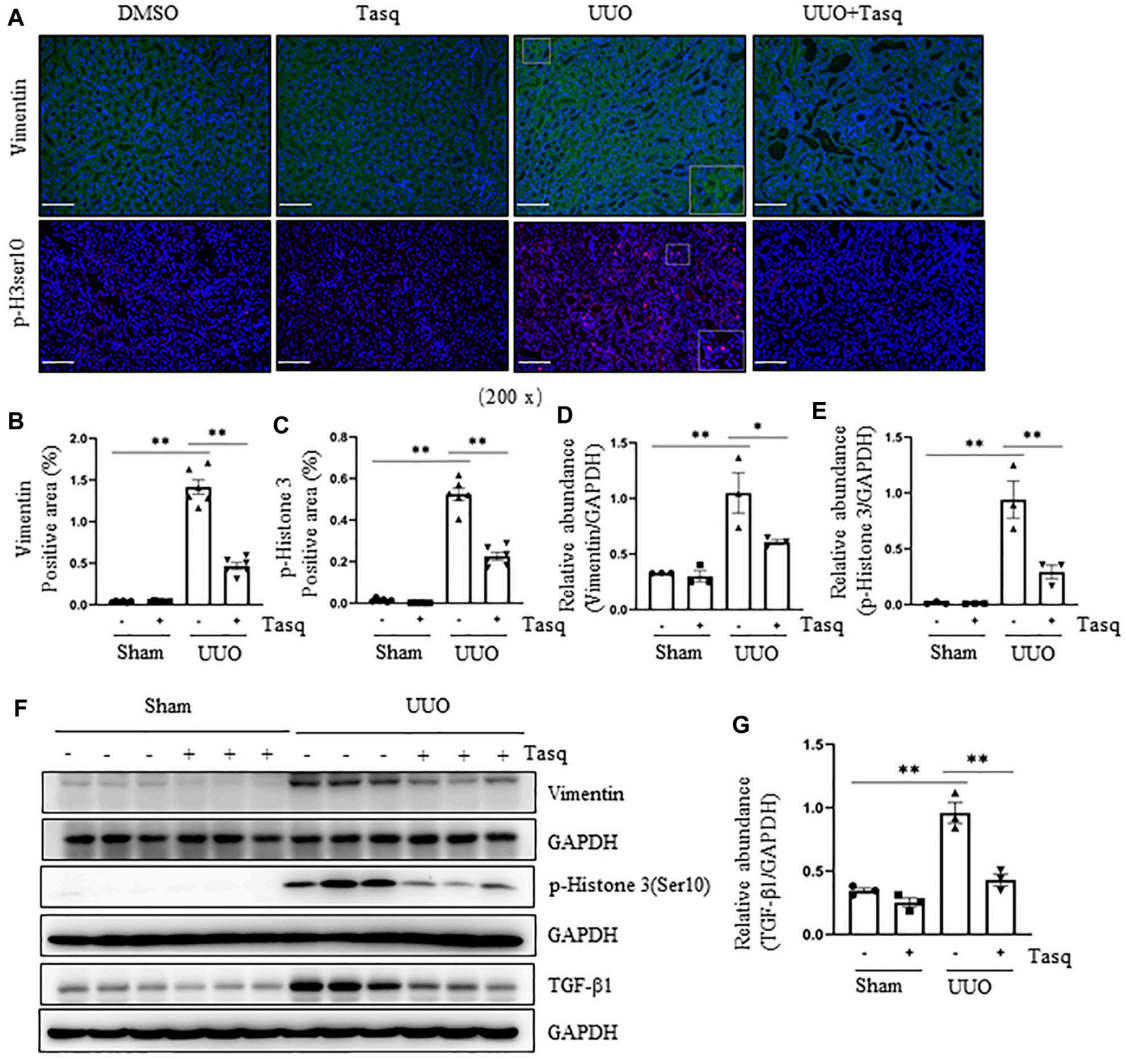
Inhibition of HDAC4 tasquinimod (Tasq) attenuates the pEMT and G2/M cell cycle arrest after ureteral obstruction. The C57BL/6 mice were subjected to sham operation or UUO and given tasquinimod or solvent after surgery and killed 7 days after surgery. **(A)** Micrographs of vimentin and p-histone H3 (Ser10) immunofluorescence staining; box at the lower right of vimentin/UUO and p-H3ser10/UUO are micrographs twice the size of the original partial position (Original magnification ×200, Scale bar = 100 μm). **(B)** HDAC4 staining shows quantitative data. **(C)** p-histone H3 (Ser10) staining shows quantitative data. Data are presented as average SE (*n* = 6). Scale bar = 100 μm. **(F)** Kidney tissue lysates from nonsurgical (Sham) and kidneys after surgery (UUO) with and without administration of tasquinimod were subjected to immunoblot analysis with antibodies against vimentin, p-Histone H3(Ser10), TGF-β1 or GAPDH. Expression levels of vimentin **(D)**, p-Histone H3 (Ser10) **(E)**, and TGF-β1 **(G)** were quantified by densitometry and normalized using GAPDH. Values are the means ± SEM of three samples. NS means no statistical significance; *p < 0.05; **p < 0.01.

Renal tubular cells following chronic injury can adopt a mesenchymal phenotype but not transform into fibroblasts (Grande et al., 2015; Lovisa et al., 2015; Huang and Susztak, 2016; Sheng and Zhuang, 2020), a process known as partial epithelial-to-mesenchymal transition (pEMT). Persistent pEMT leads to renal epithelial cell arrest in the G2/M phase of the cell cycle, resulting in the overproduction of pro-fibrotic/pro-inflammatory factors such as TGF-β1 (Yang et al., 2010). To understand whether HDAC4 contributes to this process, we first examined the effect of pharmacological and genetic inhibition of HDAC4 on the events associated with the pEMT (i. e., vimentin upregulation) and G_2_/M arrest (i.e., phosphorylation of histone H3 at serine 10 (pH3ser10) by immunostaining and immunoblotting. As shown in Figures 3A–F, the UUO injury in mice increased renal expression of vimentin and p-H3ser10, while administration of tasquinimod significantly reduced their expression. In addition, TGF-β1 was increased in the kidney of mice following UUO injury but largely suppressed by tasquinimod (Figures 3F,G). These data illustrate that HDAC4 activation is indispensable for the process of pEMT, arrest of the G_2_/M cell cycle, and subsequent production of TGF-β1 in renal epithelial cells of mice after UUO injury.

**FIGURE 4:**
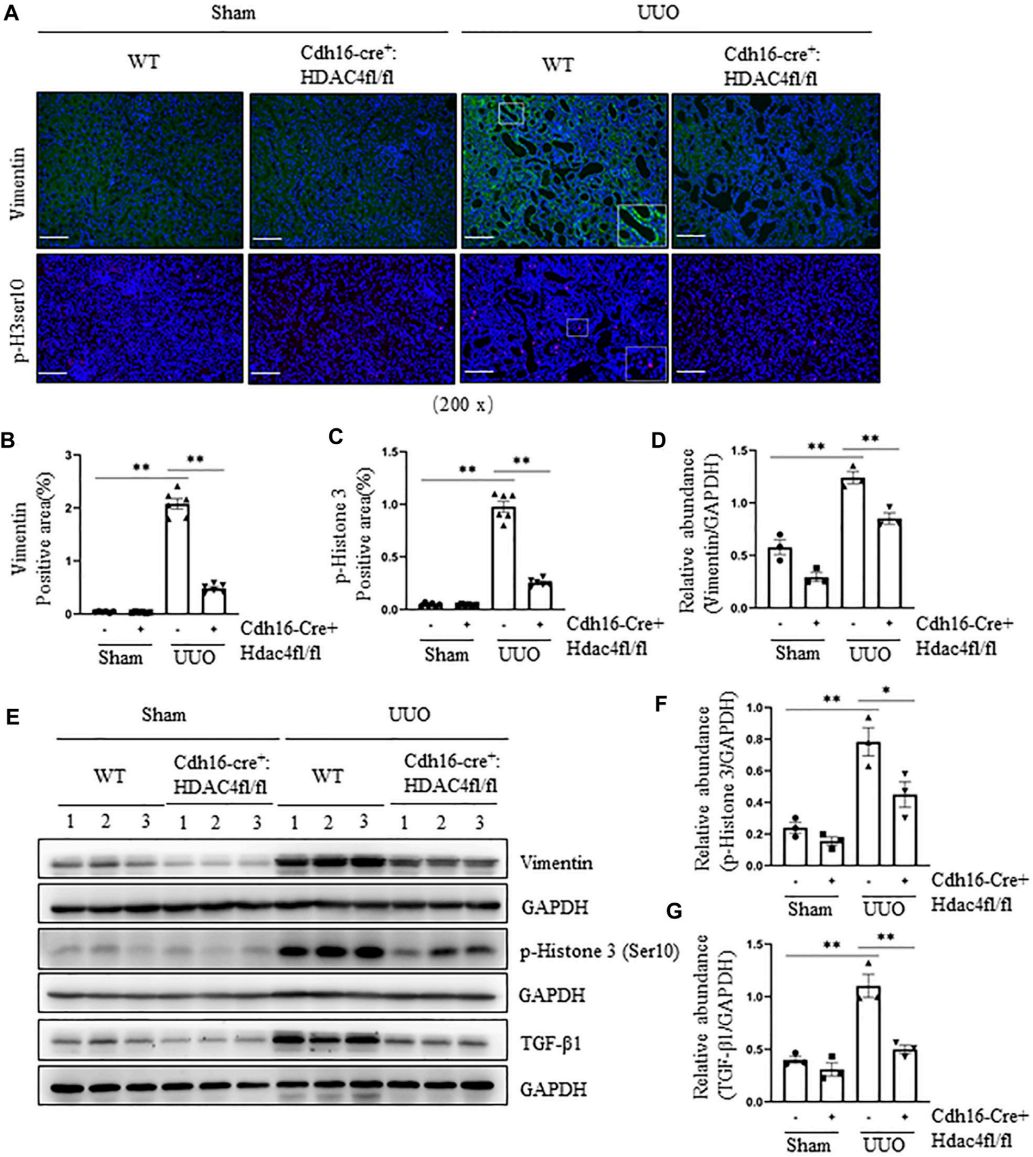
Specific deletion of HDAC4 in renal tubules attenuates the pEMT and G2/M cell cycle arrest after ureteral obstruction. Cdh16-Cre^-^: HDAC4^fl/fl^ (HDAC4-WT) and Cdh16-Cre^+^: HDAC4^fl/fl^ (HDAC4-KO) mice were killed 7 days after sham operation or UUO. **(A)** Micrographs of vimentin and p-Histone H3(Ser10) immunofluorescence staining; box at the lower right of vimentin/WT/UUO is a micrograph twice the size of the original partial position (Original magnification ×200, Scale bar = 100 μm). Vimentin staining **(B)** and p-Histone H3(Ser10) staining **(C)** show quantitative data. Data are presented as average SE (*n* = 6). Scale bar = 100 μm. **(E)** Kidney tissue lysates were subjected to immunoblot analysis with antibodies against vimentin, p-Histone H3(Ser10), TGF-β1, or GAPDH. Expression levels of vimentin **(D)**, p-Histone H3(Ser10) **(F)**, and TGF-β1 **(G)** were quantified by densitometry and normalized using GAPDH. Values are the means ± SEM of three samples. NS means no statistical significance; *p < 0.05; **p < 0.01.

To validate these observations, we further investigated the effect of conditional knockout of HDAC4 on the UUO-induced pEMT and G_2_/M cell cycle arrest by immunoblot analysis and immunofluorescence staining. Compared with the HDAC4-WT mice, the HDAC4-KO mice had much lower expression levels of vimentin and p-H3ser10 following the UUO injury (Figures 4A–F). Moreover, a lower level of renal TGF-β1 was detected in mice with HDAC4 deletion than that of the HDAC4-WT mice (Figures 4E,G). These data confirm that HDAC4 is, indeed, a critical mediator in the injury-induced pEMT and G_2_/M arrest, illustrating a fundamental mechanism by which HDAC4 contributes to renal fibrosis.

### Pharmacological and Genetic Inhibition of HDAC4 Inhibits Activation of Three Profibrotic Signaling Pathways

**FIGURE 5:**
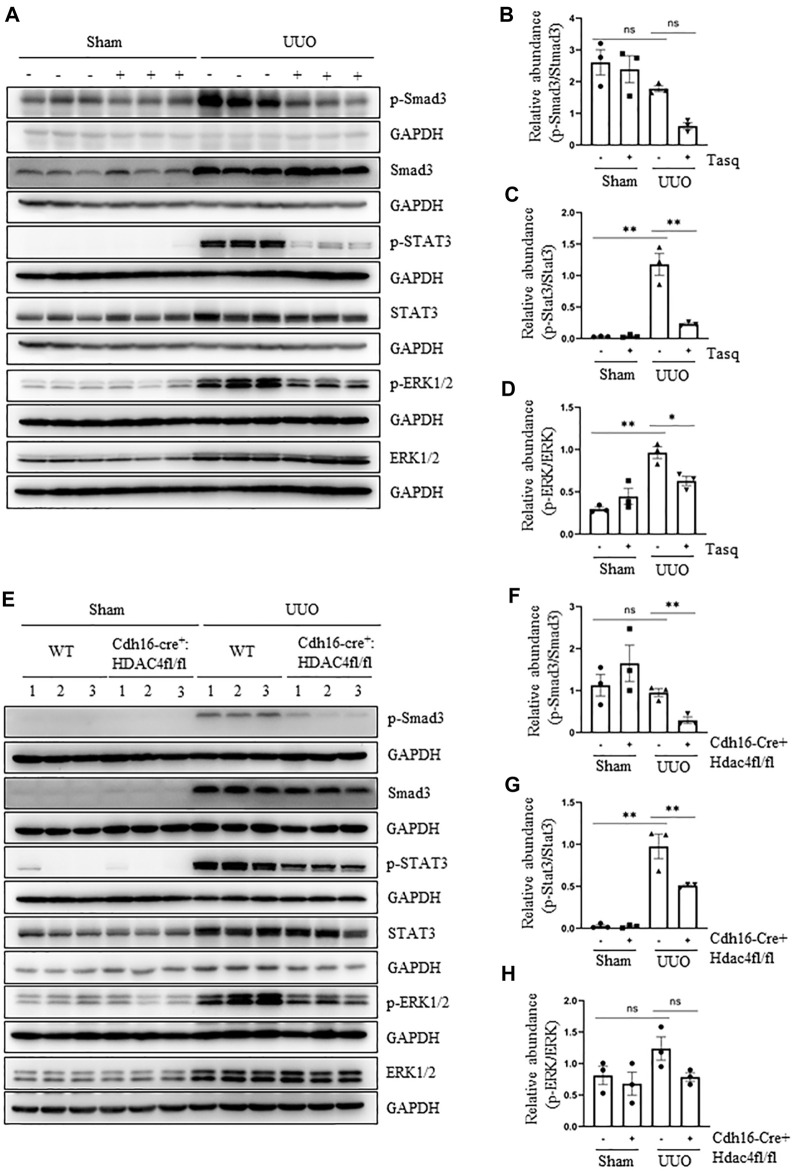
Pharmacological and genetic inhibition of HDAC4 inhibits activation of three profibrotic signaling pathways. **(A)** C57BL/6 mice were subjected to sham operation or UUO and given tasquinimod (Tasq) or solvent after surgery and killed 7 days after surgery. The kidney tissue lysates were subjected to immunoblot analysis with antibodies against p-Smad3, Smad3, p-STAT3, STAT3, p-ERK1/2, ERK1/2, or GAPDH. **(B)** Expression levels of p-Smad3 were quantified by densitometry and normalized using Smad3. **(C)** p-STAT3 was normalized with STAT3; **(D)** p-ERK1/2 was normalized with ERK1/2. **(E)** Cdh16-Cre^-^: HDAC4^fl/fl^ (HDAC4-WT) and Cdh16-Cre^+^: HDAC4^fl/fl^ (HDAC4-KO) mice were killed 7 days after sham operation or UUO. The kidney tissue lysates were subjected to immunoblot analysis with antibodies against p-Smad3, Smad3, p-STAT3, STAT3, p-ERK1/2, ERK1/2, or GAPDH. **(F)** p-Smad3 was normalized with Smad3; **(G)** p-STAT3 was normalized with STAT3; **(H)** p-ERK1/2 was normalized with ERK1/2. Values are the means ± SEM of three samples. NS means no statistical significance; *p < 0.05; **p < 0.01.

It is well known that the TGF-β_1_/Smad3 pathway is key to the development of EMT and renal fibrosis (Inazaki et al., 2004), and other signaling pathways such as STAT3 and ERK1/2 also contribute to these processes (Matsui and Meldrum, 2012; Pang et al., 2010; Bienaime et al., 2016; Andrikopoulos et al., 2019; J.; Chen et al., 2012; Rhyu et al., 2005). To elucidate whether HDAC4 would be required for the activation of these three pathways, we examined the effect of pharmacological and genetic inhibition of HDAC4 on the phosphorylation of Smad3, STAT3, and ERK1/2. The UUO injury to the kidney induced phosphorylation of Smad3, STAT3, and ERK1/2 and upregulated their total proteins. Inhibition of HDAC4, either by the specific inhibitor, tasquinimod, or by knockout, greatly reduced their phosphorylation but did not significantly alter the expression levels of total Smad3, STAT3, and ERK1/2 (Figures 5A–H). Basal levels of Smad3, STAT3, and ERK1/2 and p-Smad3 and p-ERK1/2 were detected but not affected by either tasquinimod or HDAC4 depletion (Figures 5A–H). The aforementioned results illustrate that HDAC4 may contribute to renal fibrogenesis by a mechanism associated with the activation of these three signaling pathways.

### Pharmacological and Genetic Inhibition of HDAC4 Attenuates Renal Injury and Tubular Cell Apoptosis in the Kidney Following UUO

**FIGURE 6:**
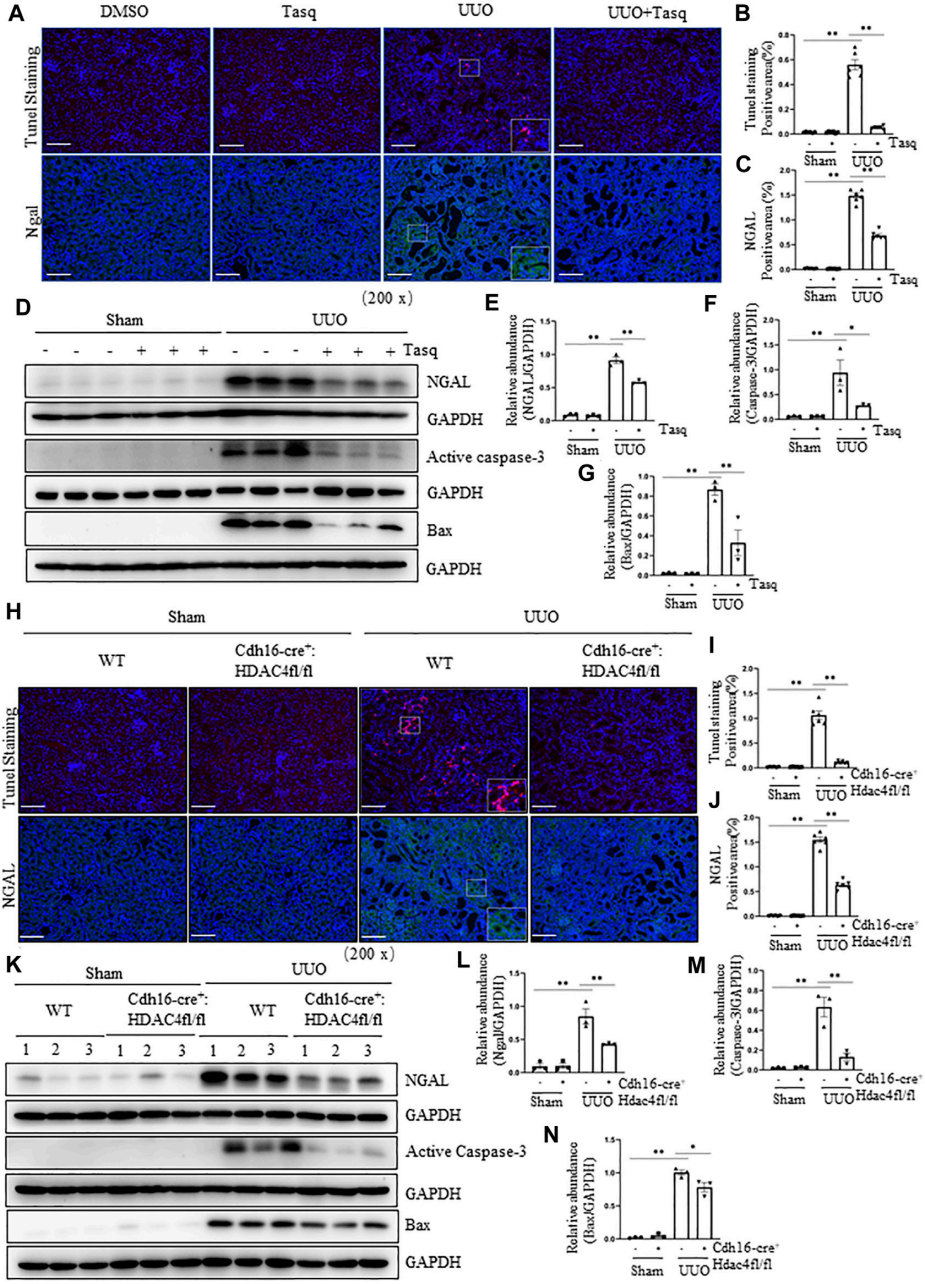
Pharmacological and genetic inhibition of HDAC4 inhibits and attenuates renal injury and tubular cell apoptosis in the kidney following UUO. **(A)** C57BL/6 mice were subjected to sham operation or UUO and given tasquinimod (Tasq) or solvent after surgery and killed 7 days after surgery. Micrographs of TUNEL and NGAL immunofluorescence staining; boxes at the lower right of TUNEL staining/UUO and NGAL/UUO are micrographs twice the size of the original partial position (Original magnification ×200, Scale bar = 100 μm). TUNEL staining **(B)** and NGAL staining **(C)** show quantitative data. Data are presented as average SE (*n* = 6). Scale bar = 100 μm. **(D)** Kidney tissue lysates were subjected to immunoblot analysis with antibodies against NGAL, active caspase-3, bax, or GAPDH. Expression levels of NGAL **(E)**, active caspase-3 **(F)**, and bax **(G)** were quantified by densitometry and normalized using GAPDH. **(H)** Cdh16-Cre^-^: HDAC4^fl/fl^ (HDAC4-WT) and Cdh16-Cre^+^: HDAC4^fl/fl^ (HDAC4-KO) mice were killed 7 days after sham operation or UUO. Micrographs of TUNEL and NGAL immunofluorescence staining; boxes at the lower right of TUNEL staining/WWT/UUO and NGAL/WT/UUO are micrographs twice the size of the original partial position (Original magnification ×200, Scale bar = 100 μm). TUNEL staining **(I)** and NGAL staining **(J)** show quantitative data. Data are presented as average SE (*n* = 6). Scale bar = 100 μm **(K)** Kidney tissue lysates were subjected to immunoblot analysis with antibodies against ngal, active caspase-3, bax, or GAPDH. Expression levels of NGALl **(L)**, active caspase-3 **(M),** and bax **(N)** were quantified by densitometry and normalized using GAPDH. Values are the means ± SEM of three samples. NS means no statistical significance; *p < 0.05; **p < 0.01.

Following UUO, renal tubular cell injury due to their expansion can lead to cell death by apoptosis (Martinez-Klimova et al., 2019). To investigate the role of HDACs in these responses, we first examined the effect of tasquinimod on the expression of NGAL, a biomarker of renal injury, and apoptosis by expression of bax and cleaved caspase 3, two apoptotic markers. Immunostaining showed that UUO injury resulted in upregulation of NGAL and increased the number of TUNEL-positive tubular cells, whereas tasquinimod treatment largely inhibited these responses (Figures 6A–E). Similarly, immunoblot analysis demonstrated the inhibitory effect of tasquinimod on the expression of NGAL and Bax and cleaved caspase 3 in the kidney subjected to UUO injury (Figures 6D–G).

Next, we investigated the effect of genetic inhibition of HDAC4 on the expression of injury and apoptosis markers. In line with the aforementioned results, deletion of HDAC4 also inhibited the expression of NGAL and reduced the number of TUNEL-positive tubular cells, as shown by immunofluorescence staining (Figures 6H–J) as well as inhibited the increase of NGAL, bax, and cleaved caspase 3 as evidenced by the immunoblot analysis in the injured kidney (Figures 6K–N).

Overall, our data illustrate that HDAC4 plays an important role in tubular cell death and fibrosis following renal injury in the mouse kidney model of UUO injury.

### Pharmacological and Genetic Inhibition of HDAC4 Inhibits Preserves Expression of Klotho

**FIGURE 7:**
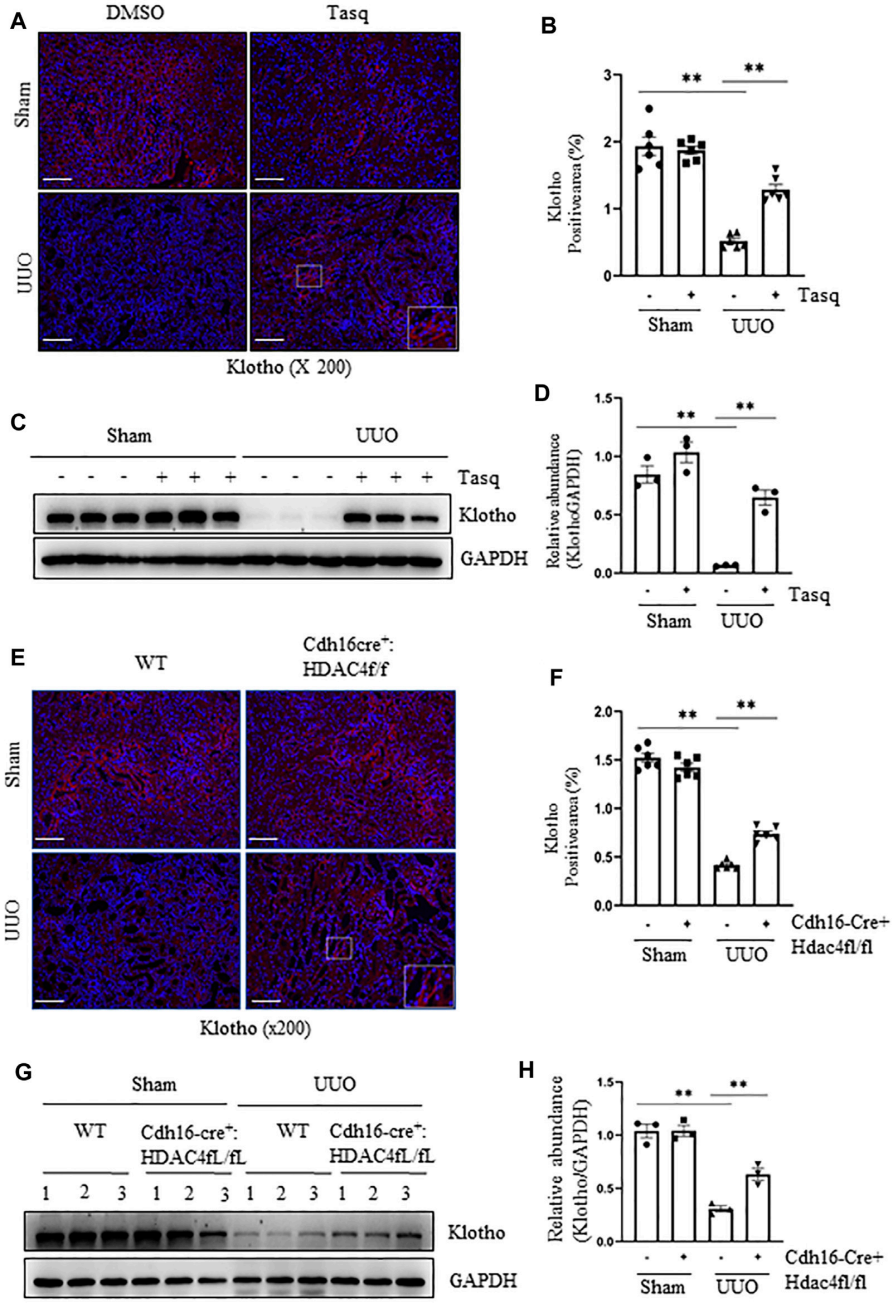
Pharmacological and genetic inhibition of HDAC4 inhibits and preserves the expression of Klotho. **(A)**) C57BL/6 mice were subjected to sham operation or UUO and given tasquinimod or solvent after surgery and killed 7 days after surgery. Micrographs of klotho immunofluorescence staining; box at the lower right of UUO/Tasq is a micrograph twice the size of the original partial position (Original magnification ×200, Scale bar = 100 μm). **(B)** Klotho staining shows quantitative data. Data are presented as average SE (*n* = 6). Scale bar = 100 μm. **(C)** Kidney tissue lysates were subjected to immunoblot analysis with antibodies against klotho or GAPDH. Expression levels of klotho **(D)** were quantified by densitometry and normalized using GAPDH. **(E)** Cdh16-Cre^-^: HDAC4^fl/fl^ (HDAC4-WT) and Cdh16-Cre^+^: HDAC4^fl/fl^ (HDAC4-KO) mice were killed 7 days after sham operation or UUO. Micrographs of klotho immunofluorescence staining, box at the lower right of UUO/Cdh16cre^+^:HDAC4f/f is a micrograph twice the size of the original partial position (Original magnification ×200, Scale bar = 100 μm). **(F)** Klotho staining shows quantitative data. Data are presented as average SE (*n* = 6). Scale bar = 100 μm. **(G)** Kidney tissue lysates were subjected to immunoblot analysis with antibodies against klotho or GAPDH. Expression levels of klotho **(H)** were quantified by densitometry and normalized using GAPDH. Values are the means ± SEM of three samples. NS means no statistical significance; *p < 0.05; **p < 0.01.

Previous studies have shown that Klotho exerts a renoprotective and an antifibrotic effect (Kale et al., 2021; Panizo et al., 2021). It is almost always repressed early after acute or chronic renal injuries, and its reduced expression is regulated *via* epigenetic modulations including acetylation (Kale et al., 2021; Xia and Cao, 2021). We thus hypothesized that HDAC4 activation would contribute to the downregulation of Klotho expression in the kidney following UUO. To test this hypothesis, we first examined the effect of HDAC4 inhibition on the expression of Klotho in the kidney with or without UUO. Immunostaining showed that compared with the basal level of Klotho in normal mice, its expression in the kidney of UUO mice was significantly reduced, whereas treatment with either tasquinimod or deletion of HDAC4 largely restored the Klotho expression (Figures 7A,B,E,F). Immunoblot analysis also showed that either tasquinimod treatment or HDAC4 knockout in part preserved Klotho expression in the kidney of mice in response to UUO injury (Figures 7C,D,G,H).

## Discussion

A growing number of studies have revealed that HDACs are widely involved in the development of various kidney diseases (Zhou et al., 2021; N.; Liu and Zhuang, 2015). We and others have provided evidence that among class IIa HDACs isoforms, HDAC4 is most commonly expressed in renal tubular cells of a murine model of fibrosis induced by UUO injury (Xiong et al., 2019) and podocytes in a murine model of diabetic nephropathy and human diabetic kidneys (Wang et al., 2014). In this study, we demonstrate that pharmacological and genetic inhibition of HDAC4 attenuates renal fibrosis and suppresses the molecular mechanisms leading to renal fibrosis, including the pEMT, fibroblast activation, and activation of the TGF-β1/Smad3, STAT3, and ERK1/2 signaling pathways. Moreover, HDAC4 inhibition reduces renal injury and tubular cell apoptosis and preserves the expression of Klotho, a potent renoprotective protein. On this basis, we suggest that HDAC4 is a critical mediator of renal fibrosis and a potential therapeutic target for the treatment of CKD.

CKD as a whole is characterized by glomerular sclerosis, tubular atrophy, and interstitial fibrosis (Venkatachalam et al., 2015; Fraser and Roderick, 2019). In the UUO model, it is characterized by tubular atrophy and interstitial fibrosis (Venkatachalam et al., 2015; Fraser and Roderick, 2019). Interstitial fibrosis includes interstitial fibroblast activation, brought on by interstitial fibroblast activation, myofibroblast transformation, inflammatory response, and deposition of ECM components (Ruiz-Ortega et al., 2020). α-SMA is a hallmark of myofibroblasts, and fibronectin is one of the major ECM proteins (F. Liu and Zhuang, 2019). Our data showed that treatment with tasquinimod to selectively inhibit HDAC4 or specific deletion of HDAC4 in renal tubular cells largely attenuated the development of fibrosis as seen with Masson’s trichrome staining and reduced expression of α-SMA and fibronectin in the UUO mouse kidney, suggesting that HDAC4 plays an important role in mediating the activation of renal interstitial fibroblasts and overproduction of ECM proteins following injury. Since tasquinimod was administrated 24 h after UUO when renal fibroblasts were supposed to be activated already, our results further suggest that HDAC4 not only mediates renal fibrosis but also contributes to its progression. Therefore, administration of HDAC4 inhibitors may improve outcomes after acute injury and slow progression to CKD. In agreement with our speculation, Wang et al. (2014) have reported that peritoneal injection of siRNA specific to HDAC4 could also suppress podocyte injury and ameliorate diabetic nephropathy in a murine model. Given that diabetic nephropathy is characterized by glomerular sclerosis and renal interstitial fibrosis and HDAC4 is also highly expressed in podocytes, it would be reasonable to speculate that treatment with tasquinimod would be effective in attenuating diabetic nephropathy and other kidney diseases due to glomerular sclerosis and interstitial fibrosis.

The molecular mechanisms leading to renal interstitial fibrosis and glomerular sclerosis are incompletely defined. Most insults to the kidney can damage tubular cells, resulting in maladaptive repair, atrophy, and pEMT when the injury is severe or persistent (Ferenbach and Bonventre, 2015; Docherty et al., 2019; Sheng and Zhuang, 2020). The pEMT is a state in which renal tubular cells lose their epithelial features and acquire a mesenchymal phenotype that reexpresses vimentin, a protein mostly expressed in embryonic kidneys. These cells remained attached to the basement membrane and do not transform into fibroblasts (Lovisa et al., 2015; Huang and Susztak, 2016). Our previous study and other studies showed that pEMT occurs in the kidney following UUO injury, while treatment with MC1568, a general HDAC IIa inhibitor, attenuates renal fibrosis and reduces the expression of α-SMA, fibronectin, and collagen I. (Xiong et al., 2019). MC1568 and HDAC4 siRNA do the same in cultured renal epithelial cells (Xiong et al., 2019). These data suggest the importance of class IIa HDACs, in particular HDAC4, in controlling the development of pEMT. In the current study, by using both HDAC4 selective inhibitor and genetic depletion of it in renal tubular cells, we provide further evidence that HDAC4 is a critical mediator in the pEMT of renal tubular cells in response to injury. In addition, renal epithelial cells with pEMT are frequently arrested at the G2/M phase of the cell cycle, becoming a profibrotic phenotype that generates and releases a large amount of profibrotic and proinflammatory factors. These factors stimulate the transformation of fibroblasts to myofibroblasts and proinflammatory responses in the tubulointerstitium (Sheng and Zhuang, 2020). We, therefore, examined the effect of tasquinimod and HDAC4 depletion on the expression of p-H3ser (10) and TGF-β1. Our results show that both pharmacological and genetic inhibition of HDAC4 dramatically reduces the expression of these two molecules, indicating the role of HDAC4 in regulating these processes. It is likely that HDAC4 only in part contributes to these two processes since HDAC4 blockade led to an incomplete inhibition of the expression of p-H3ser (10) and TGF-β1. In addition, since cdh16 cre is only expressed in collecting ducts and part of proximal tubules (Shao et al., 2002a; Shao et al., 2002b), HDAC4 may contribute to the aforementioned responses due to incomplete deletion of HDAC4 in renal tubules. Although HDAC4 inhibition also reduced the expression of α-SMA in the injured kidney, we do not speculate that HDAC4 directly mediates the activation of renal interstitial fibroblasts. Instead, HDAC4 may indirectly stimulate the transformation of resident fibroblasts/pericytes to myofibroblasts by promoting the formation of a profibrotic/secretory phenotype of renal epithelial cells and release of some profibrotic factors into the interstitium. In support of this statement, we observed that HDAC4 was only expressed in renal tubular cells of the injured kidneys (Xiong et al., 2019), and inhibition of HDAC4 reduced the expression of TGF-β1.

Overproduction of TGF-β1 and subsequent activation of the TGF-β1/Smad3 pathway is the most important mechanism for various insults to induce renal fibrosis and promote the progression of CKD to ESRD (Meng et al., 2016; Gu et al., 2020). Activation of the TGF-β1/Smad3 pathway promotes not only pEMT but also the transformation of fibroblasts to myofibroblasts (Meng et al., 2016; Gu et al., 2020). As discussed above, HDAC4 may indirectly activate renal interstitial fibroblasts by inducing pEMT and releasing TGF-β1 (Sheng and Zhuang, 2020). In addition, we found in cultured renal epithelial cells that HDAC4 promotes TGF-β1–stimulated EMT and expression of fibronectin and collagen I and III (Xiong et al., 2019). These data, together with our results showing that HDAC4 inhibition reduced the expression of TGF-β1 in the UUO injured kidney, suggest that HDAC4 may regulate renal fibrosis through diverse mechanisms. Nevertheless, HDAC4-mediated regulation of profibrotic signaling may not be limited to the TGF-β1/Smad3 pathway since we found that pharmacological and genetic inhibition of HDAC4 also reduced phosphorylation of STAT3 and ERK1/2, two pathways that also regulate multiple profibrotic processes (i.e., renal fibroblast activation, EMT, and inflammation) and lead to renal fibrosis.

It is well known that renal fibrosis results from unchecked repair of renal tubular cells (Ferenbach and Bonventre, 2015; Sheng and Zhuang, 2020). However, the degree of injury determines the fate of renal tubular cells and whether they undergo complete repair or suffer from maladaptive repair, leading to chronic injury. In addition to its role in controlling the events leading to renal fibrosis, HDAC4 may also contribute to the injury and death of renal tubular cells. This hypothesis was supported by our observations that deletion of HDAC4 or administration of the HDAC4 inhibitor at 24 h of UUO reduced the expression of NGAL and Bax and cleaved caspase-3. As we utilized an approach of non-inducible deletion of HDAC4 in this study, genetic deletion of HDAC4 and pharmacological inhibition interrupt the early and late phases of the process, leading to renal tubular cell injury and death. Our results indicate that HDAC4 plays a role in initiating renal epithelial cell injury and death during the course of renal fibrogenesis. Given that either genetic deletion of HDAC4 or late treatment with tasquinimod led to inhibition of renal injury and fibrosis, it remains obscure that the antifibrotic effect of HDAC4 inhibition is primarily due to prevention of renal tubular cell injury or inhibition of renal fibrosis. It will be interesting to address this issue in our future studies.

Currently, it remains unclear how HDAC4 mediates the molecular events leading to tubular cell injury/death. Klotho, a protein that is predominantly expressed in renal tubular cells, protects against acute and chronic kidney injury caused by various insults, including ischemia/reperfusion, chemical agents, infection, and obstruction (Doi and Masaki, 2017; Neyra and Hu, 2017; Xia and Cao, 2021). However, Klotho is frequently repressed early, after acute or chronic renal injuries, and its levels inversely correlate with disease progression and severity (Doi and Masaki, 2017; Neyra and Hu, 2017; Xia and Cao, 2021). Thus, preservation and/or restoration of the Klotho expression during the course of injuries is critical for its protective effects. Although the Klotho expression is regulated by multiple mechanisms, epigenetic regulation, especially acetylation, has been shown to be involved in this process. Cao et al. demonstrated that administration of a pan HDAC inhibitor, trichostatin A (TSA), alleviated Klotho repression in a murine model of CKD triggered by feeding an adenine-containing diet. Klotho loss prevention by TSA is associated with increased promoter-associated histone acetylation, elevating Klotho transcription (Lin et al., 2017). Additional studies showed that UUO-induced loss of Klotho in murine fibrotic kidneys and expression of renal fibrosis–associated proteins were attenuated by genistein administration *via* a mechanism associated with inhibiting histone 3 deacetylation of the Klotho promoter, suggesting that Klotho restoration can occur *via* epigenetic histone acetylation. At present, it remains unknown which HDAC IIa isoforms play a role in this process. Our studies showed that pharmacological and genetic inhibition of HDAC4 enhanced acetylation of histone H3, coincident with the restoration of the Klotho expression. As such, HDAC4 may be a critical mediator in regulating the depression of Klotho during the acute phase and perhaps chronic phase of renal injury following UUO. Since Klotho functions as a coreceptor of FGF receptors (FGFRs) to activate a specific fibroblast growth factor 23 (FGF23) signal pathway and FGF23 exerts its biological effects in a Klotho-dependent manner (Lu and Hu, 2017; Navarro-Garcia et al., 2021), it is possible that HDAC4-mediated regulation of Klotho may also indirectly influence the biological functions of FGF23 such as mineral homeostasis and CKD-related bone disorders and cardiovascular problems. Furthermore, an investigation is needed to address these issues.

In summary, this study was the first to demonstrate the importance of HDAC4 in mediating the development and progression of renal fibrosis in a murine model of kidney injury following UUO. HDAC4-induced renal fibrosis was associated with renal tubular cell injury and apoptosis, promotion of pEMT, and preserving the Klotho expression at transcriptional levels. On this basis, it is believed that inhibition of HDAC4 would be a new therapeutic strategy for the clinical treatment of fibrotic kidney disease.

## Data Availability

The original contributions presented in the study are included in the article/Supplementary Material; further inquiries can be directed to the corresponding author.
